# FAM201A, a long noncoding RNA potentially associated with atrial fibrillation identified by ceRNA network analyses and WGCNA

**DOI:** 10.1186/s12920-022-01232-w

**Published:** 2022-04-11

**Authors:** Xi Chen, Xiang-Yu He, Qing Dan, Yang Li

**Affiliations:** 1 Department of Geriatrics, The First Affiliated Hospital of Chongqing Medical University, Chongqing, China; 2 Department of Ophthalmology, The 958th Hospital, Southwest Hospital, Third Military Medical University (Army Medical University), Chongqing, China; 3 Department of Cardiology, General Hospital of Chinese People’s Liberation Army, No. 28 Fu Xing Road, Beijing, 100853 China

**Keywords:** Atrial fibrillation, Competing endogenous RNA, Weighted gene co-expression network analysis, Long noncoding RNA family with sequence similarity 201-member A

## Abstract

**Background:**

Being the most common arrhythmia in clinic, atrial fibrillation (AF) causes various comorbidities to patients such as heart failure and stroke. LncRNAs were reported involved in pathogenesis of AF, yet, little is known about AF-associated lncRNAs. The present study aims to explore lncRNAs associated with AF susceptibility based on competing endogenous RNA (ceRNA) network analysis and weighted gene co-expression network analysis (WGCNA).

**Methods:**

GSE41177 and GSE79768 datasets were obtained from the Gene Expression Omnibus (GEO) database. Competing endogenous RNA (ceRNA) network analysis was performed using GSE41177. Differentially expressed lncRNAs (DElncRNAs), mRNAs (DEmRNAs) between AF patients and patients with sinus rhythm (SR) were identified from GSE41177 using R software. Then, the ceRNA network was constructed based on DElncRNAs, the predicted target miRNAs and DEmRNAs. Weighted gene co-expression network analysis (WGCNA) was performed using GSE79768 to validate the AF-related lncRNAs identified from GSE41177. LncRNA modules and crucial lncRNAs relevant to AF and were identified.

**Results:**

In summary, 18 DElncRNAs and 350 DEmRNAs were found between AF patients and SR patients. A total of 5 lncRNAs, 10 miRNAs, and 21 mRNAs were contained in the final ceRNA network. Taking into consideration both the ceRNA theory and inference scores from the comparative toxicogenomics database (CTD) database, the ceRNA axis FAM201A-miR-33a-3p-RAC3 was identified as mostly relevant to AF susceptibility. FAM201A (Gene significance, GS = − 0.62; Module membership, MM = 0.75) was also proved in the blue module, which was identified most highly relevant with AF by WGCNA.

**Conclusions:**

These results demonstrated that decreased expression of FAM201A might be associated with susceptibility of AF. Working as the ceRNA to regulate RAC3 might be one function of FAM201A in AF susceptibility, which requires further exploration in future research.

**Supplementary Information:**

The online version contains supplementary material available at 10.1186/s12920-022-01232-w.

## Background

Atrial fibrillation (AF), the most common cardiac arrhythmia, affects approximately 34 million people worldwide, and the number increases with aging [1, 2]. Being a major contributor to stroke, heart failure, sudden death and myocardial infarction, AF poses a significant burden to patients and society [2]. The clinical risk factors of AF include gender, alcohol consumption, smoking, obesity and some clinical comorbidities [3]. Besides, a number of protein-coding genes have been identified significantly associated with risk of AF. Though, only a small proportion of heritability for AF has been uncovered [4, 5]. Until now, the pathogenesis of AF still remains poorly understood, limiting the discovery of novel therapeutic targets for AF.

Long noncoding RNAs (lncRNAs), with more than 200 nucleotides in length, are non-protein-coding RNAs, taking an important part in transcriptional and epigenetic gene regulation [6]. Recently, a large number of studies have demonstrated that lncRNAs are involved in pathogenesis of various diseases, such as cancers, diabetes, heart failure and myocardial infarction [7–10]. More importantly, some lncRNAs were proved to be new biomarkers or therapeutic targets for AF, such as lncRNA HNRNPU-AS1, lncRNA PVT1, lncRNA GAS5 [11–13]. Still, little is known about lncRNAs associated with AF susceptibility, which deserves further discoveries.

One pivotal regulatory function of lncRNAs is working as competing endogenous RNA (ceRNA) to regulate mRNA transcription. It was stated in this ceRNA hypothesis that lncRNA could compete for miRNA via shared microRNA response elements, and subsequently sponge miRNA to indirectly regulate mRNA expression [14]. In the pathogenesis of AF, for example, lncRNA PVT1, which was increased in atrial samples of AF patients, acted as a sponge for miR-128-3p and up-regulated Sp1 expression to facilitate atrial fibrosis [12]. LncRNA TCONS-00106987 acted as a sponge for miR-26 and up-regulated KCNJ2 expression to trigger atrial electrical remodeling [15]. Thus, analyses of ceRNA networks turned out to be an efficient method to discover AF-related lncRNAs.

Besides, another biology algorithm, weighted gene co-expression network analysis (WGCNA) [16], can be used to analyze crucial genes or novel biomarkers of various diseases including AF [17]. Briefly, gene modules were constructed and associated with clinical traits to identify disease related modules and the key genes in each module. It is worth noting that not only protein-coding RNAs, but also non-protein-coding RNAs could be used to construct the co-expression networks associated with diseases [18, 19].

For short of AF-related lncRNA microarray datasets, combination of diverse methods or biology algorithms are deadly required to deeply discover lncRNAs involved in pathogenesis of AF. Therefore, in the present study, we aim to identify lncRNAs associated with AF susceptibility based on ceRNA network analyses, as well as WGCNA.

## Materials and methods

### Microarray data collection

The lncRNA and mRNA expression profiles of two datasets were retrieved from the Gene Expression Omnibus (GEO) database (https://www.ncbi.nlm.nih.gov/geo/). The dataset GSE41177 was used to identify lncRNAs associated with AF susceptibility based on ceRNA network construction, and GSE79768 was used to validate the potential role of lncRNAs for AF based on WGCNA. GSE41177 (platform: GPL570) contains left atrial appendages and paired pulmonary vein and the surrounding left atrial junctions from 16 AF patients and 3 patients with sinus rhythm (SR). GSE79768 (platform: GPL570) contains left atrial appendages and right atrial appendages from 7 AF patients and 6 SR patients. Data of GSE41177 were obtained from AF and SR patients undergoing valvular heart disease surgery. LA-PV junction specimens were taken from the area between the right-superior and right-inferior PVs adjacent to the atriotomy site, as described elsewhere [20]. Data of GSE79768 were obtained from patients receiving surgery for mitral valve or coronary artery disease, as described elsewhere [21]. In the present study, to achieve the sample consistency of the two datasets, only data of left atrial appendages in GSE41177 and GSE79768 were used for analyses. Thus, a total of 23 left arial appendages of AF patients and 9 left arial appendages of SR were included in the study. Additionally, there is evidence that gene expression files of left atrial and right atrial are not usually identical and left atrial is proved more pivotal in AF initiation and maintenance [22, 23].

### Differential lncRNA and mRNA expression analyses

The lncRNA and mRNA expression profiles were retrieved from dataset GSE41177 for differential expression analyses and ceRNA network construction. Identification of differentially expressed lncRNAs (DElncRNAs), mRNAs (DEmRNAs) between AF patients and SR patients were performed using the “limma” package of R software (Version 3.6.3) [24]. The adjusted *P* value < 0.05 and |log2-fold change (FC)|> 1 was taken as the threshold to select DElncRNAs and DEmRNAs. Hierarchical cluster heatmaps were generated to represent expression intensity and direction of DElncRNAs and DEmRNAs using the “pheatmap” package of R software based on Euclidean distance.

### Construction of the ceRNA network

DElncRNAs associated with target miRNAs were predicted using the miRcode database (http://www.mircode.org/), which contains the putative interactions between lncRNAs and miRNAs [25]. Then, these miRNAs associated target mRNAs were predicted based on the miRDB (http://mirdb.org), miRTarBase (http://miRTarBase.cuhk.edu.cn/), and TargetScan (http://www.targetscan.org) databases [26–28]. Only those identified in all three databases were selected as target mRNAs. The overlap of target mRNAs and DEmRNAs, together with DElnRNAs and predicted miRNAs, were obtained to construct the final ceRNA network using cytoscape software (Version 3.7.2) [29].

### Gene ontology enrichment analyses for mRNAs in the ceRNA network

To reveal the potential biological functions of mRNAs in the ceRNA network, Gene Ontology (GO) enrichment analyses was performed using the “clusterProfiler” package of R software [30]. The bar graph was generated to display the enrichment results. The threshold was set as the adjusted *P* value < 0.05.

### Potential AF susceptibility lncRNAs and mRNAs prediction

The Comparative Toxicogenomics Database (CTD) (http://ctd.mdibl.org) database contains data of associations between chemicals, gene products, phenotypes, diseases, and environmental exposures [31]. The CTD database was used to predict the potential AF-related lncRNAs and mRNAs in the ceRNA network, with the inference score reflecting the association between AF and lncRNAs or mRNAs.

### Validation for potential role of lncRNAs through WGCNA

The lncRNA expression profiles were retrieved from dataset GSE79768 for WGCNA. All 1210 lncRNAs were selected to construct a co-expression network using the “WGCNA” package in R software[16]. A proper soft threshold of 9 was chosen to satisfy the degree of independence of 0.85 with the minimum value. An adjacency matrix was constructed and converted into a topological overlap matrix to reflect the correlation strength in the co-expression network. Hierarchical clustering was performed using DynamicTreeCut algorithm to construct the network modules with the minimum module size of 30 and height cutoff of 0.25. The module eigengene (ME) is the first principal component of the module, representing the overall expression profiles of each module. To identify modules relevant to AF susceptibility, the correlation between MEs and atrial rhythm phenotypes were analyzed with Pearson’s correlation and visualized by the heatmap. A threshold of *P* < 0.05 was used to screen the modules significantly associated with AF susceptibility. Gene significance (GS) is defined as the correlation between each gene and the clinical trait. Module membership (MM) is defined as the correlation between each gene and each module eigengene. The lncRNAs satisfying the criteria of |GS|> 0.6 and |MM|> 0.5 were identified as crucial lncRNAs relevant to AF.

## Results

### Differential expression analyses of lncRNAs and mRNAs in atrial fibrillation

**Fig. 1:**
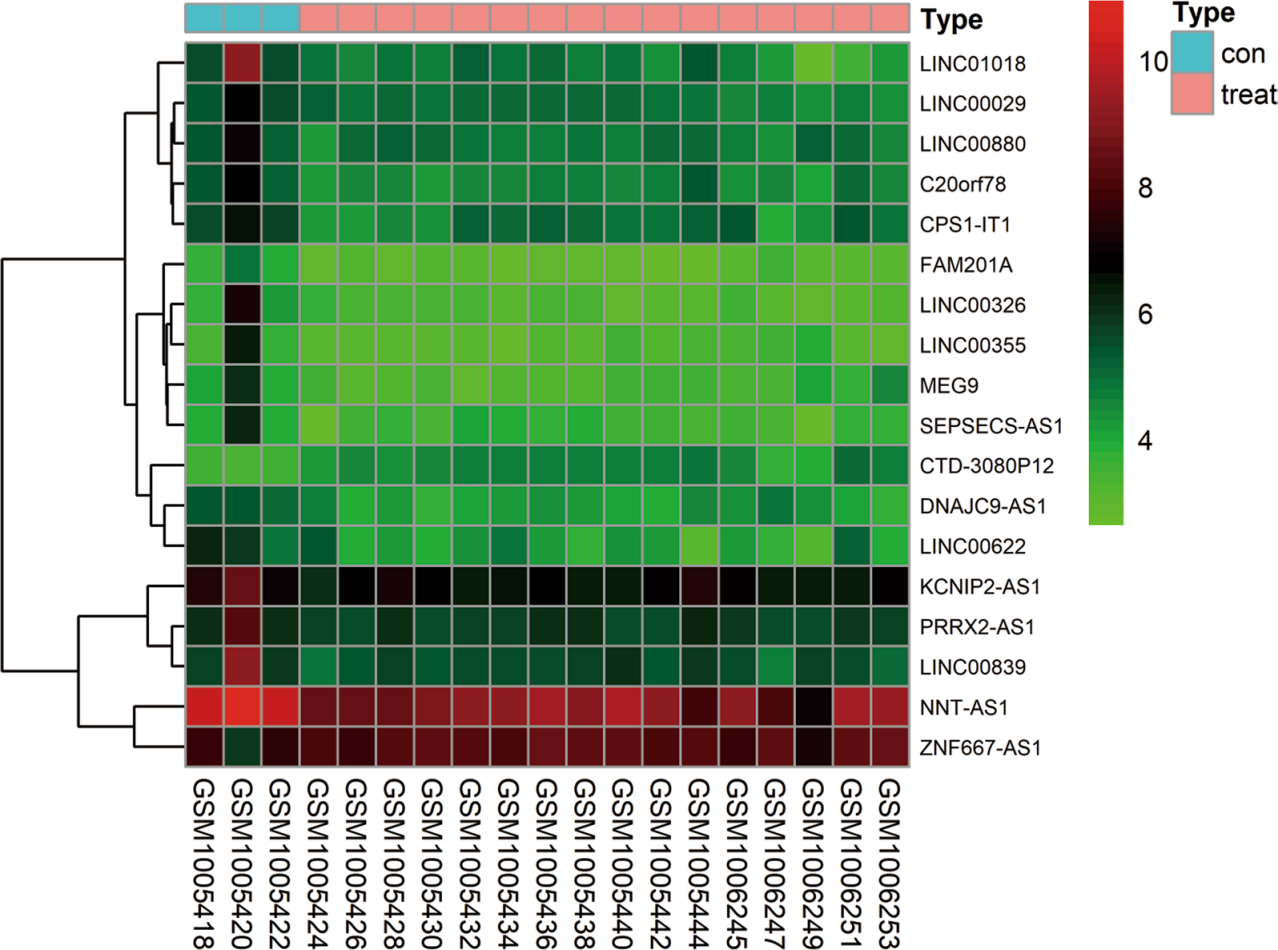
Heatmap of differentially expressed lncRNAs in AF and SR samples. The vertical axis represents samples, and the horizontal axis represents lncRNAs. Pink color represented AF samples, and blue color represented SR samples. Green color indicates down-regulated expression levels, and red color indicates up-regulated expression levels

Microarray data of GSE41177 were downloaded and used for analyses. A total of 18 DElncRNAs (Additional file 1) and 350 DEmRNAs (Additional file 2) were identified between AF patients and SR patients, using the threshold of an adjusted *P* value < 0.05 and |log2 FC|> 1 as cut-off. The volcano plots showed all RNA expression levels in AF samples compared to SR samples (Additional file 3). The heatmap showed the distinguished expression levels of lncRNAs (Fig. 1) and mRNAs (Additional file 4) between AF and SR groups.

### Construction of the ceRNA network

**Fig. 2:**
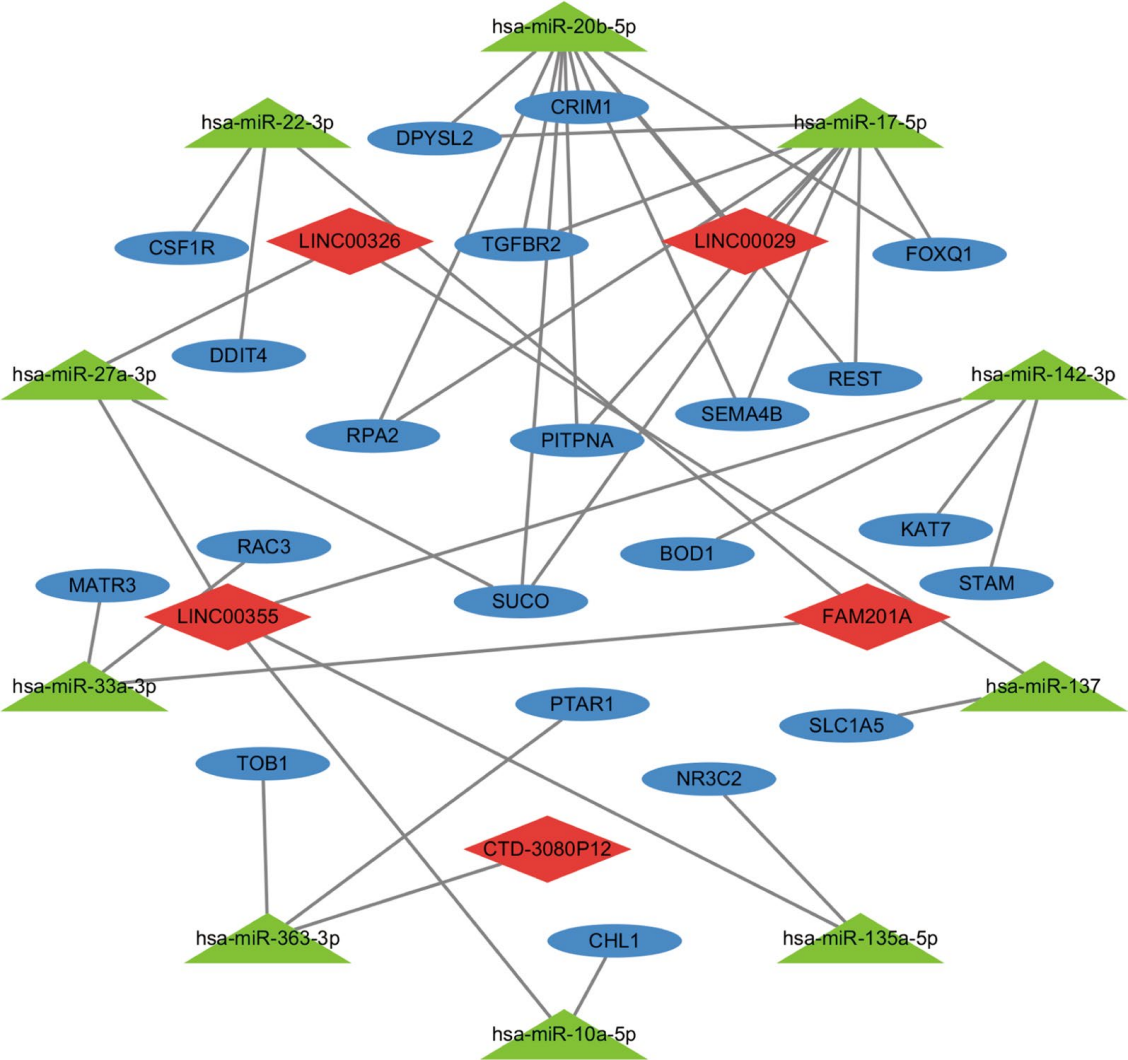
Competing endogenous RNA network of lnRNA-miRNA-mRNA. Diamonds represent lncRNAs, trianglse represent miRNAs and oval represent mRNAs

We predict the miRNAs that interact with DElncRNAs based on miRcode database. A total of 222 interactions between 6 DElncRNAs and 157miRNAs were identified. Then the target mRNAs of abovementioned 157 miRNAs were predicted based on the miRDB, miRTarBase, and TargetScan databases. Overall, 992 mRNAs identified in all three databases were selected as target mRNAs. The overlap of these 992 mRNAs and 350 DEmRNAs were used to construct the ceRNA network, which finally contained a total of 5 lncRNAs, 10 miRNAs, and 21 mRNAs (Fig. 2, Tables 1 and 2, Additional file 5). Except for one up-regulated lncRNA CTD-3080P12, the other four lncRNAs (FAM201A, LINC00326, LINC00029, LINC00355) were all down-regulated in AF patients compared with SR patients.

**Table 1:** Differentially expressed lncRNAs in the ceRNA network

	logFC	AveExpr	t	*P* Value	adj. *P* val	B
FAM201A	− 1.17	3.18	− 5.97	6.51 × 10^–6^	3.58 × 10^–3^	3.97
CTD-3080P12	1.09	4.40	5.50	1.89 × 10^–5^	5.14 × 10^–3^	2.98
LINC00326	− 1.86	3.52	− 4.69	1.25 × 10^–4^	1.14 × 10^–2^	1.22
LINC00029	− 1.00	5.01	− 4.62	1.51 × 10^–4^	1.20 × 10^–2^	1.05
LINC00355	− 1.33	3.42	− 3.65	1.51 × 10^–3^	3.38 × 10^–2^	− 1.10

**Table 2:** Differentially expressed mRNAs in the ceRNA network

	logFC	AveExpr	t	*P* value	adj. *P* val	B
SLC1A5	1.09	6.89	3.30	3.45 × 10^–3^	4.89 × 10^–2^	− 1.86
REST	− 1.20	7.10	− 5.80	9.58 × 10^–6^	4.00 × 10^–3^	3.62
PITPNA	1.11	8.21	5.03	5.67 × 10^–5^	8.47 × 10^–3^	1.96
RAC3	− 1.12	5.13	− 3.47	2.32 × 10^–3^	4.09 × 10^–2^	− 1.50
FOXQ1	− 1.03	4.34	− 5.89	7.71 × 10^–6^	3.67 × 10^–3^	3.82
CSF1R	1.75	9.00	3.66	1.48 × 10^–3^	3.35 × 10^–2^	− 1.08
BOD1	1.99	8.69	3.47	2.29 × 10^–3^	4.07 × 10^–2^	− 1.48
RPA2	2.29	8.54	3.61	1.65 × 10^–3^	3.49 × 10^–2^	− 1.18
DPYSL2	2.37	11.37	3.56	1.85 × 10^–3^	3.69 × 10^–2^	− 1.29
DDIT4	1.09	10.54	4.41	2.50 × 10^–4^	1.53 × 10^–2^	0.58
KAT7	− 1.19	8.58	− 4.41	2.45 × 10^–4^	1.51 × 10^–2^	0.60
TOB1	− 1.38	10.11	− 3.64	1.55 × 10^–3^	3.40 × 10^–2^	− 1.12
NR3C2	− 1.43	8.25	− 5.90	7.56 × 10^–6^	3.67 × 10^–3^	3.84
SEMA4B	− 1.05	7.32	− 4.35	2.88 × 10^–4^	1.63 × 10^–2^	0.44
CHL1	− 1.07	5.03	− 3.42	2.56 × 10^–3^	4.32 × 10^–2^	− 1.59
CRIM1	− 1.25	8.94	− 4.33	2.96 × 10^–4^	1.64 × 10^–2^	0.42
TGFBR2	1.20	8.89	3.31	3.32 × 10^–3^	4.81 × 10^–2^	− 1.83
SUCO	− 1.46	8.13	− 3.73	1.23 × 10^–3^	3.12 × 10^–2^	− 0.91
STAM	− 1.07	8.60	− 4.48	2.11 × 10^–4^	1.42 × 10^–2^	0.74
MATR3	− 1.40	4.07	− 3.35	3.08 × 10^–3^	4.68 × 10^–2^	− 1.76
PTAR1	− 1.01	6.88	− 3.29	3.52 × 10^–3^	4.94 × 10^–2^	− 1.88

### Gene ontology enrichment analyses for mRNAs in the ceRNA network

The results of GO enrichment analyses with the screening criteria of adjusted *P* value < 0.05 were presented in Additional file 6 and Additional file 7. The mRNAs in the ceRNA network were mainly enriched in transmembrane receptor protein kinase activity, protein phosphatase binding, phosphatase binding, transmembrane receptor protein tyrosine kinase activity. Neither specific function nor specific pathway was identified in the enrichment analyses. The reason for that was speculated to be the small number of mRNAs enrolled in the enrichment analyses.

### Potential AF associated lncRNAs and mRNAs prediction in the ceRNA netwrok

LncRNAs are supposed to positively regulate mRNAs by working as miRNA sponges based on ceRNA theory. In our ceRNA network, expression direction of seven down-regulated lncRNA and mRNA pairs were in accord with the ceRNA theory, including two DElncRNAs (FAM201A, LINC00355) and seven DEmRNAs (RAC3, MATR3, NR3C2, KAT7, CHL1, SUCO, STAM). Then, the CTD database was used to predict the potential role of above DElncRNAs and DEmRNAs in AF. Inference scores were used to reflect the associations between AF and above RNAs. Finally, one lncRNA and seven mRNAs including FAM201A (Inference score: 3.08), NR3C2 (Inference score: 24.69), RAC3 (Inference score: 22.57), KAT7 (Inference score: 16.61), STAM (Inference score: 9.62), MATR3(Inference score: 5.27), CHL1 (Inference score: 3.77), and SUCO (Inference score: 1.79) turned out to have potential associations with AF. Regarding mRNAs, NR3C2 and RAC3 have relatively higher inference scores for AF. Taking inference scores of both lncRNAs and mRNAs into consideration, the ceRNA axis FAM201A-miR-33a-3p-RAC3 was predicted to have potential role for AF susceptibility.

### Validation for potential role of lncRNAs through WGCNA

**Fig. 3:**
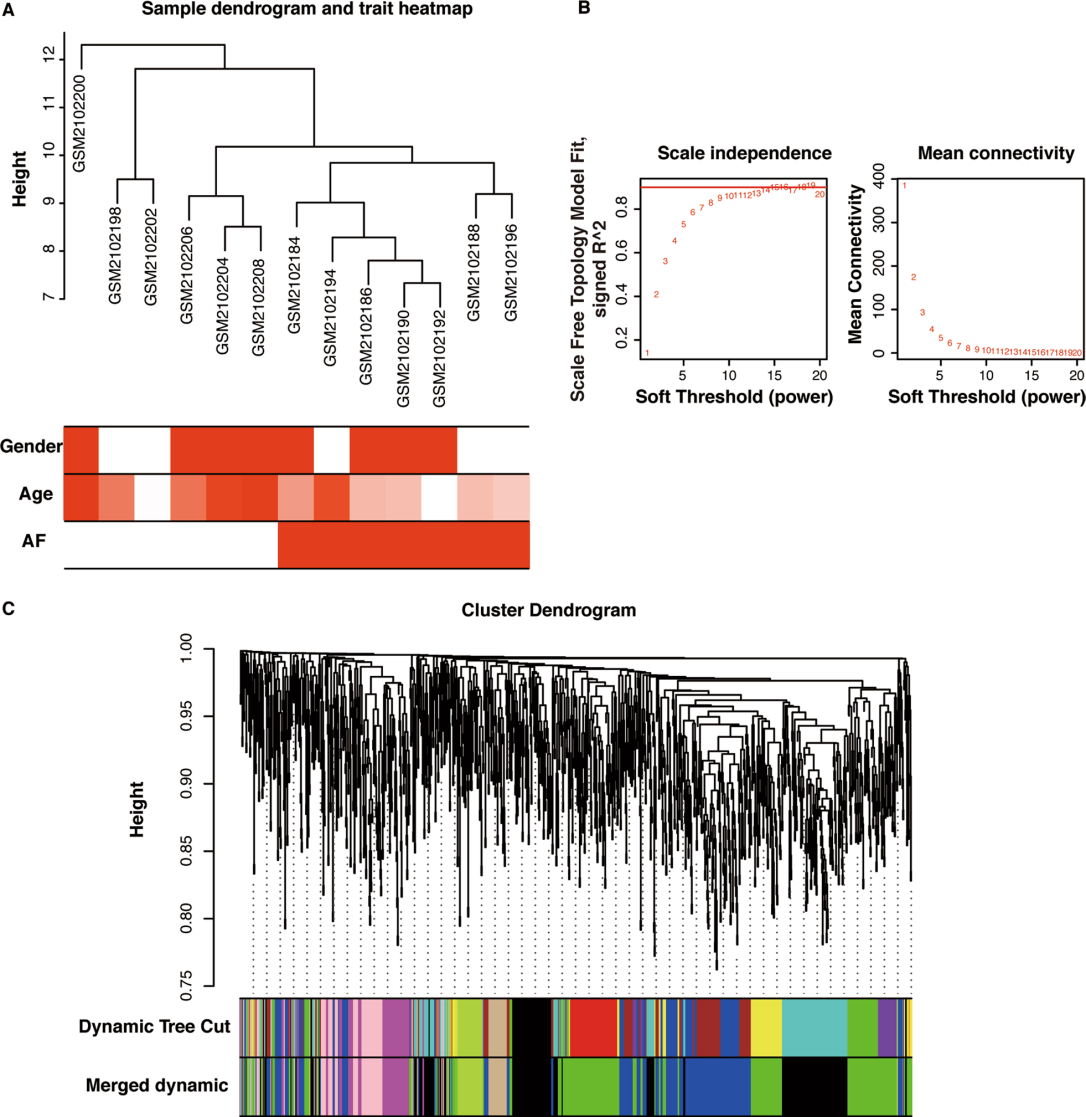
Construction of weighted co‐expression network. **a** The sample clustering tree and trait heatmap in 13 samples. **b** Soft-threshold power analysis. **c** Co-expression cluster dendrogram. Each color represents one specific module by WGCNA

WGCNA was performed to further validate the potential role of lncRNAs in AF. All 1210 lncRNA expression matrices from GSE7976 were screened for construction of the co-expression network. The sample clustering tree and trait heatmap was illustrated in Fig. 3a. The soft‐threshold of 9 was set to construct a scale-free network, with the scale‐free topology fit index > 0.85 (Fig. 3b). The final eight modules identified based on average hierarchical clustering and dynamic tree cutting were shown in Fig. 3c.

**Fig. 4:**
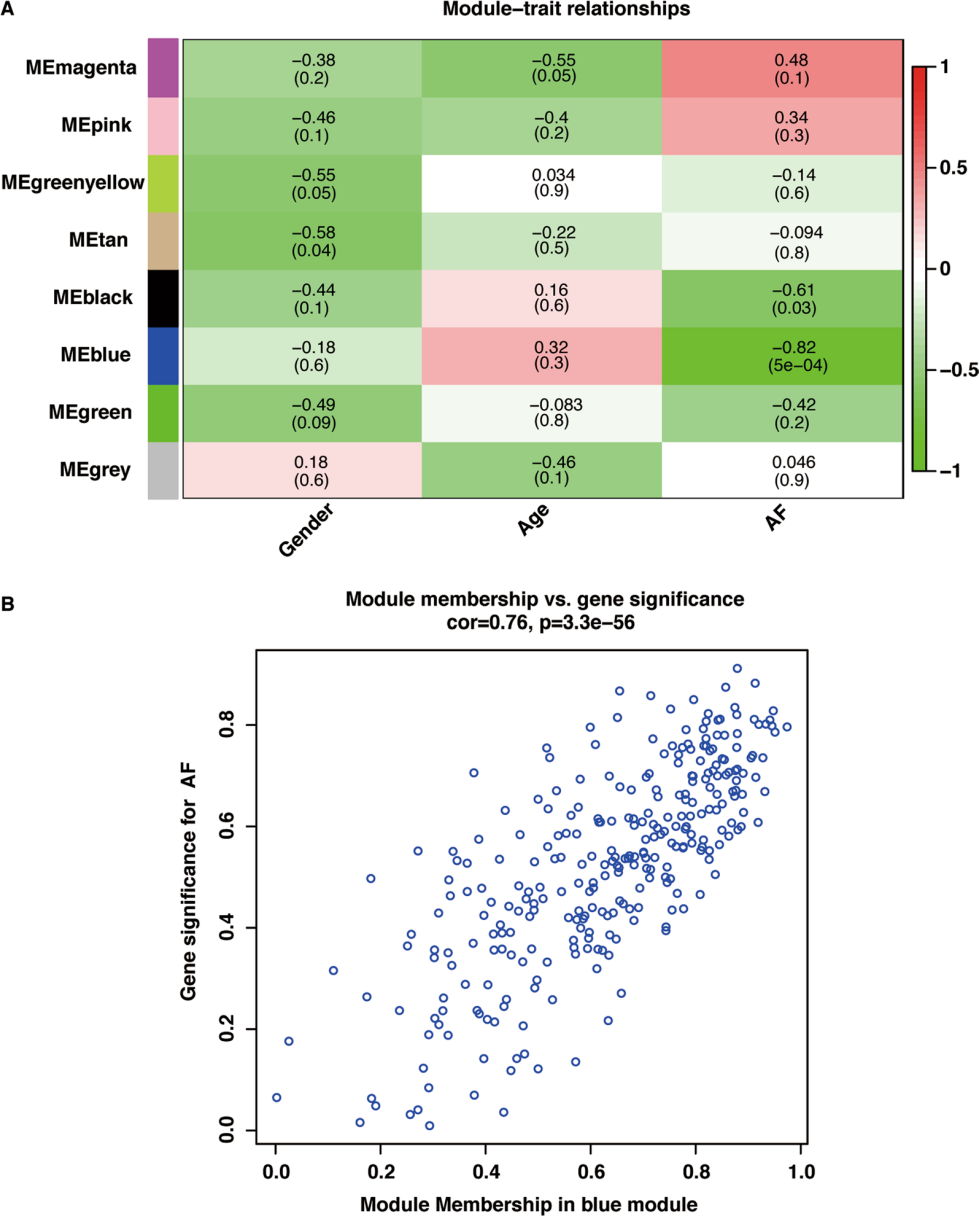
Identification of AF associated module. **a** Heatmap of the correlation between the module eigengenes and AF. **b** Correlation between MM and GS for AF in blue module

The module‐trait association analysis was performed by calculating the correlation between MEs and the AF phenotype. Blue module (r = − 0.82; *P* = 5 × 10^–4^) and black module (r = − 0.61; *P* = 0.03) were highly relevant with AF (Fig. 4a). The significant correlation was observed between MM and GS for AF in the blue module, as shown in Fig. 4b. A total of 95 lncRNAs in blue module were identified as crucial lncRNAs associated with AF, according to the criteria of |GS|> 0.6 and |MM|> 0.5 (Additional file 8). We mainly focused on the MM and GS for AF of lncRNAs, namely, FAM201A, LINC00355, LINC00326, LINC00029, LINC00355, CTD-3080P12, which were identified in the ceRNA network. Finally, FAM201A (GS: − 0.62, P.GS: 0.02; MM: 0.75, P.MM: 3.35 × 10^−3^) and LINC00326 (GS: − 0.60, P.GS: 0.03; MM: 0.78, P.MM: 1.58 × 10^−3^) were in the blue module and proved to be highly related to AF (Table 3).

**Table 3:** MM and GS for AF of lncRNAs in the ceRNA network

lncRNA	GS	P.GS	MM	P.MM	Module color
FAM201A	− 0.62	0.02	0.75	3.35 × 10^–3^	Blue
LINC00355	− 0.20	0.51	0.02	0.96	Greenyellow
CTD-3080P12	0.05	0.87	0.25	0.41	Pink
LINC00326	− 0.60	0.03	0.78	1.58 × 10^–3^	Blue
LINC00029	0.37	0.21	0.10	0.75	Magenta

*AF* atrial fibrillation, *GS* gene significance, *MM* module membership

Taken together, these results demonstrated that FAM201A might have great potential for susceptibility of AF based on the ceRNA network, CTD database and WGCNA. FAM201A may function, at least partly, as ceRNA to regulate RAC3 in AF susceptibility.

## Discussion

In the present study, 23 left arial appendages of AF patients and 9 left atrial appendages of SR patients were enrolled from two datasets, one for screening AF-related lnRNAs by ceRNA network analyses and another for validation by WGCNA. By construction of a ceRNA network, combined with a CTD database, the ceRNA axis FAM201A-miR-33a-3p-RAC3 was identified associated with AF susceptibility. Subsequently, by WGCNA for lncRNAs, two co-expression lncRNA modules were proved associated with AF and FAM201A was finally validated to be highly negatively related to AF. Collectively, lncRNA FAM201A was speculated to function, at least partly, as ceRNA to regulate RAC3 in AF susceptibility.

FAM201A refers to the lncRNA family with sequence similarity 201-member A, located in genomic 9p13.1 with 2.9 Kbp long [32]. FAM201A was demonstrated to be involved in various diseases previously, especially cancers. In patients with lung squamous cell cancer, FAM201A was up-regulated. By regulating ATP-binding cassette transporter E1, FAM201A participated in cell proliferation, migration, invasion and influenced the survival of these patients [33]. In tissues from non-small cell lung cancer patients, elevated expression level of FAM201A was detected related to radioresistance. For these patients, FAM201A-miR-370-EGFR was suggested as a key axis in regulation of radiotherapy sensitivity [34]. Another in vivo experiment showed that high level expression of FAM201A involved in development of lung adenocarcinoma and down-regulation of FAM201A exerted the opposite effect [35]. In addition to cancers, down-regulation of FAM201A was reported to be associated with Osteonecrosis of the femoral head through bioinformatics analysis and quantitative real-time polymerase chain reaction experiments [36]. For the first time, FAM201A was revealed to be associated with AF susceptibility in the present study. In order to confirm the potential function of FAM201A on AF, both the ceRNA network analyses and WGCNA were applied. Identical results concerning the function of FAM201A on AF were achieved based on the two different methods and two different microarray datasets. Thus, these reliable results indicated that down-regulation of FAM201A may serve as potential prediction of AF susceptibility.

The underlying mechanism of FAM201A on AF could be elucidated, at least partly, through ceRNA network analyses. In our constructed ceRNA network, FAM201A regulated RAC3 by sponging miR-33a-3p. Down-regulation of FAM201A and the consequent down-regulation of RAC3 were detected to correlate with AF susceptibility. RAC3 refers to the Rac family of small guanosine triphosphatases [37]. This is the first time that alteration of RAC3 expression from left atrial appendages of AF patients compared with SR patients has been reported. However, expression level of RAC3 was previously reported to be elevated in leukocytes from AF patients compared with controls [38]. Actually, there is a contradiction between their study and the present study. It was believed that the different samples obtained might be responsible for the discrepancy between the findings from this previous study and our study. As stated above, different samples obtained for research could contribute to distinct gene expression [23].

Although we have not confirmed through experiments that FAM201A plays the role of ceRNA and regulates RAC3 in AF susceptibility. There was evidence that the ceRNA axis FAM201A-miR-33a-3p-RAC3 might increase AF susceptibility through autophagy. The fact is that FAM201A, miR-33a-3p and RAC3 all correlate with autophagy, directly or indirectly. He et al. reported that in RAC3 knockdown human umbilical vein endothelial cells, level of autophagy was detected to be much higher, which was related to inhibition of endothelial dysfunction caused by oxidized low‐density lipoprotein. The results indicated the role of RAC3 on endothelial dysfunction by down-regulating autophagy [39]. Rubio et al. demonstrate that the expression level of RAC3 was negatively associated with autophagy, influencing chemoresistance of colorectal cancer [40]. In a word, RAC3 was verified to have a capacity for down-regulating autophagy. Han et al. found that in primary hepatocellular cancer, the down-regulation of miR-33a-3p expression is related to metastases and poor prognosis, and confirmed that increased miR-33a-3p expression could inhibit cell migration and invasion [41]. A large number of studies have confirmed that decreased level of cell migration and invasion are often significantly related to increased level of autophagy [42, 43]. It can be seen that the increased expression of miR-33a-3p is indirectly related to the increase of autophagy. One of the main functions of FAM201A is to regulate cell proliferation and invasion. Specifically, low expression of FAM201A can inhibit cell proliferation and invasion [35]. There is evidence that decreased cell proliferation and invasion are related to autophagy activation [44, 45]. Therefore, the decrease of lncRNA FAM201A expression may be indirectly related to the increase of autophagy. Interestingly, high levels of autophagy was observed in both AF patients and rabbit models of atrial rapid pacing [46]. Promotion of autophagy by overexpression of autophagy-related gene7 could lead to decreased L-type calcium channel, diminished L-type calcium current, abbreviated action potential duration, and higher AF incidence. It was also testified that up-regulated autophagy could aggravate the autophagic degradation of L-type calcium channels and the related electrical remodeling in AF [46]. In addition to these findings, the high level of autophagy or autophagy-related gene expression were also observed elsewhere in experimental and clinical AF [47, 48]. Thus, we speculated based on the findings of our study that decreased expression of RAC3 resulting from decreased FAM201A might potentially promote autophagy and the consequent vulnerability to AF. We would indeed elucidate this mechanism in the future experiments.

There were still some limitations in the present study. First, the sample size in our study was not large enough. Although we testified the role of lncRNAs through different methods, more validations were still needed to confirm the role of some key lncRNAs in AF. Second, the expression levels of lncRNAs in the dataset used for WGCNA were relatively much lower than those of protein-coding genes. Much data about lnRNAs identified in the ceRNA network would be missed if we include all protein-coding genes in the analyses. Therefore, in order to obtain as much information about lncRNAs as possible, we performed WGCNA only for lnRNAs, which would inevitably lead to the missing information about lncRNA-mRNA interactions. Third, the GO enrichment analyses failed to provide us significant clues on the underlying mechanisms of AF related to genes identified in the present study, which could be explained by the small number of mRNAs included in the final ceRNA network used for this enrichment analyses. Most importantly, the underlying mechanism of FAM201A related to AF was speculated on previous research and our bioinformatics analysis, and further experiments were badly required to verify this theory, in order to better explain the mechanisms of AF.

## Conclusions

In conclusion, we identified an important ceRNA axis FAM201A-miR-33a-3p-RAC3 associated with AF susceptibility through analyses of the ceRNA network. The pivotal role of FAM201A on AF was then validated by WGCNA. These findings indicated that decreased expression of FAM201A exerted an important role on AF susceptibility through down-regulating RAC3 and gave us a novel clue on further experiments about the underlying mechanisms of AF.

## Supplementary Information

**Additional file 1**. Differentially expressed lncRNAs in AF samples compared with SR samples.

**Additional file 2**. GO enrichment of DEmRNAs.

**Additional file 3**. Volcano plot of all RNA expression levels in AF samples compared to SR samples.

**Additional file 4**. Heatmap of differentially expressed mRNAs in AF and SR samples.

**Additional file 5**. Bar plot of GO enrichment of DEmRNAs.

**Additional file 6**. Differentially expressed mRNAs in AF samples compared with SR samples.

**Additional file 7**. The association between lncRNAs and miRNAs, miRNA and mRNAs in the ceRNA network.

**Additional file 8**. Crucial lncRNAs in blue module associated with AF based on WGCNA.

## Data Availability

The datasets (accession number: GSE41177; accession number: GSE79768) used in the present study are available in the GEO database. The dataset GSE41177 can be accessed in the website: https://www.ncbi.nlm.nih.gov/geo/query/acc.cgi?acc=GSE41177. The dataset GSE79768 can be accessed in the website: https://www.ncbi.nlm.nih.gov/geo/query/acc.cgi?acc=GSE79768.

## Abbreviations

AF: Atrial fibrillation
SR: Sinus rhythm
CeRNA: Competing endogenous RNA
WGCNA: Weighted gene co-expression network analysis
GEO: Gene expression omnibus
DElncRNAs: Differentially expressed lncRNAs
DEmRNAs: Differentially expressed mRNAs
GO: Gene ontology
CTD: Comparative toxicogenomics database
LncRNA: Long noncoding RNA
ME: Module eigengene
GS: Gene significance
MM: Module membership
FAM201A: Family with sequence similarity 201-member A
RAC3: Rac family of small guanosine triphosphatases
