# Supplementary figures and images for: FAM201A, a long noncoding RNA potentially associated with atrial fibrillation identified by ceRNA network analyses and WGCNA
Source: BMC Med Genomics. 2022 Apr 11;15:80. doi: 10.1186/s12920-022-01232-w (PMC8996407; doi:10.1186/s12920-022-01232-w)

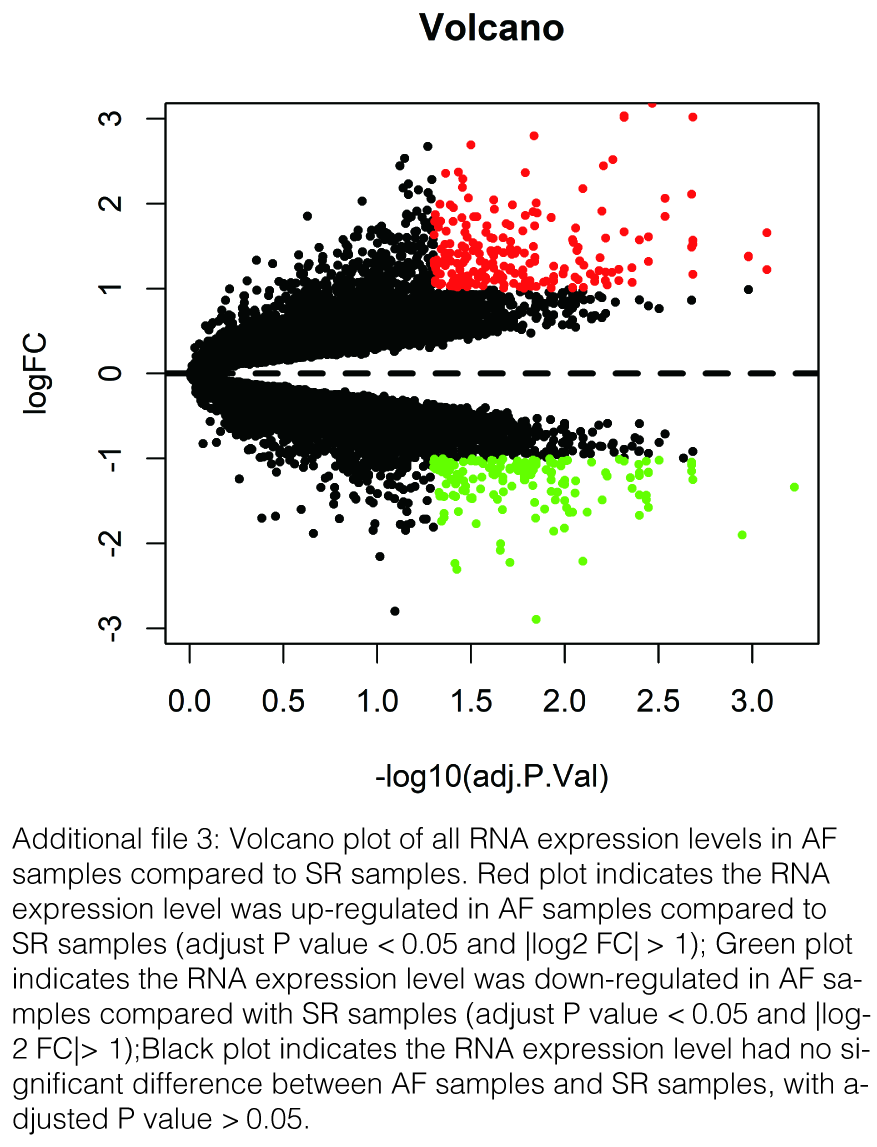

Supplement: Supplementary file 3 — Additional file 3. Volcano plot of all RNA expression levels in AF samples compared to SR samples. [file 12920_2022_1232_MOESM3_ESM.tif]

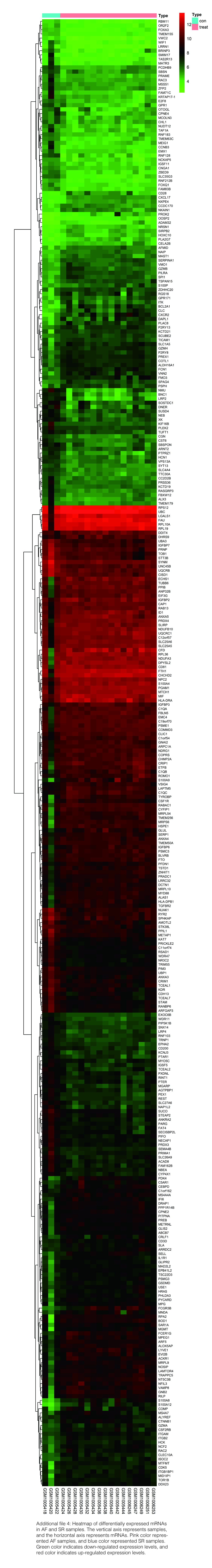

Supplement: Supplementary file 4 — Additional file 4. Heatmap of differentially expressed mRNAs in AF and SR samples. [file 12920_2022_1232_MOESM4_ESM.tif]

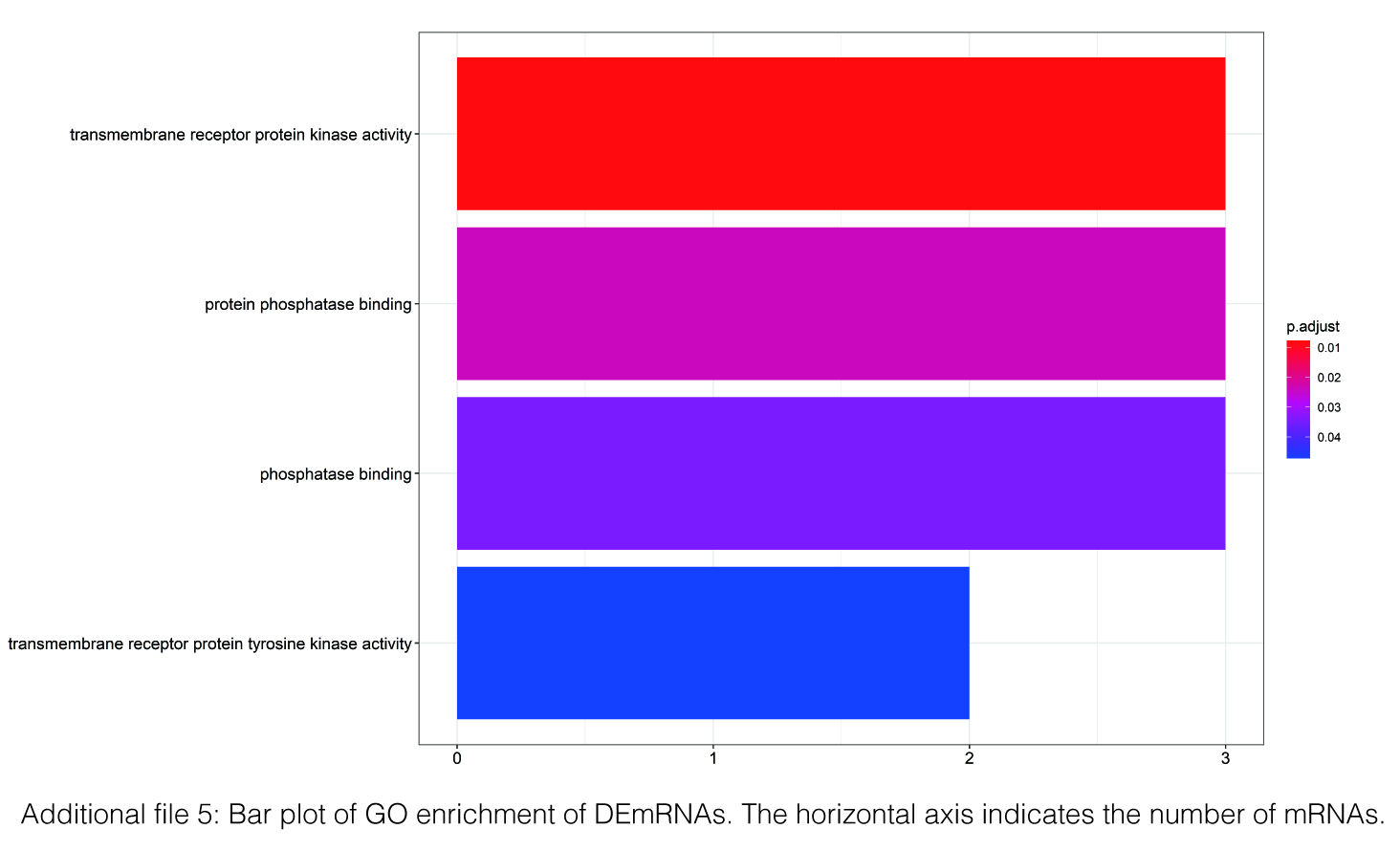

Supplement: Supplementary file 5 — Additional file 5. Bar plot of GO enrichment of DEmRNAs. [file 12920_2022_1232_MOESM5_ESM.tif]
